# Critical windows of greenness exposure during preconception and gestational periods in association with birthweight outcomes

**DOI:** 10.1088/2752-5309/ad0aa6

**Published:** 2023-11-16

**Authors:** Zhenchun Yang, Jiawen Liao, Yi Zhang, Yan Lin, Yihui Ge, Wu Chen, Chenyu Qiu, Kiros Berhane, Zhipeng Bai, Bin Han, Jia Xu, Yong Hui Jiang, Frank Gilliland, Weili Yan, Zhanghua Chen, Guoying Huang, Junfeng (Jim) Zhang

**Affiliations:** 1 Duke Global Health Institute, Duke University, Durham, NC, United States of America; 2 Department of Population and Public Health Sciences, Keck School of Medicine, University of Southern California, Los Angeles, CA, United States of America; 3 Children’s Hospital of Fudan University, Shanghai Key Laboratory of Birth Defect, Shanghai, People’s Republic of China; 4 Department of Biostatistics, Mailman School of Public Health, Columbia University, New York, NY, United States of America; 5 State Key Laboratory of Environmental Criteria and Risk Assessment, Chinese Research Academy of Environmental Sciences, Beijing, People’s Republic of China; 6 Department of Genetics, Neuroscience, and Pediatrics, Yale University School of Medicine, New Haven, CT, United States of America; 7 Division of Environmental Science and Policy, Nicholas School of the Environment, Duke University, Durham, NC, United States of America

**Keywords:** greenness, NDVI, air pollutant, preconception, pregnancy, birthweight, urbanicity

## Abstract

Few studies have examined the association between greenness exposure and birth outcomes. This study aims to identify critical exposure time windows during preconception and pregnancy for the association between greenness exposure and birth weight. A cohort of 13 890 pregnant women and newborns in Shanghai, China from 2016–2019 were included in the study. We assessed greenness exposure using Normalized Difference Vegetation Index (NDVI) during the preconception and gestational periods, and evaluated the association with term birthweight, birthweight *z*-score, small-for-gestational age, and large-for-gestational age using linear and logistic regressions adjusting for key maternal and newborn covariates. Ambient temperature, relative humidity, ambient levels of fine particles (PM_2.5_) and nitrogen dioxide (NO_2_) assessed during the same period were adjusted for as sensitivity analyses. Furthermore, we explored the potential different effects by urbanicity and park accessibility through stratified analysis. We found that higher greenness exposure at the second trimester of pregnancy and averaged exposure during the entire pregnancy were associated with higher birthweight and birthweight *Z*-score. Specifically, a 0.1 unit increase in second trimester averaged NDVI value was associated with an increase in birthweight of 10.2 g (95% CI: 1.8–18.5 g) and in birthweight *Z*-score of 0.024 (0.003–0.045). A 0.1 unit increase in an averaged NDVI during the entire pregnancy was associated with 10.1 g (95% CI: 1.0–19.2 g) increase in birthweight and 0.025 (0.001–0.048) increase in birthweight *Z*-score. Moreover, the associations were larger in effect size among urban residents than suburban residents and among residents without park accessibility within 500 m compared to those with park accessibility within 500 m. Our findings suggest that increased greenness exposure, particularly during the second trimester, may be beneficial to birth weight in a metropolitan area.

**Abbreviations**
CI: Confidence interval
OR: Odds ratio
NDVI: Normalized Difference Vegetation Index
PB: Preterm birth
SES: Socioeconomic status

## 1. Introduction

Developing countries have experienced rapid urbanization over the past decades. For example, the urbanization rate in China increased drastically from about 18% in 1978 to more than 60% in 2018 (Zhao *et al* 2022). Urbanization results in rapid changes in land use in urban and adjacent suburban areas, with more land used for construction and transportation and less open spaces and parks for people to contact with natural environmental elements. One strategic approach to building healthy cities is ensuring green space access (World Health Organization 2020). By definition, green space means land that is partly or completely covered with grass, trees, shrubs, or other vegetation, which includes parks, community gardens and cemeteries (EPA 2023). Exposure to green space has been associated with a range of health benefits (Twohig-Bennett and Jones 2018, Yang *et al* 2021), including reduced diastolic blood pressure, lower salivary cortisol level (reduced stress level), lower heart rate, decreased incidences of diabetes, all-cause and cardiovascular mortality, and improved mental health. Green space can benefit human health through the pathways of (1). Promoting physical activity, (2). Providing psychosocial influences such as increased social interactions; (3). Directly decreasing psychological stress and depression; and (4). Mitigating air pollution, noise, and heat exposure (Franchini and Mannucci 2018).

During pregnancy, living in a green area can potentially benefit health through promoting physical activities, mitigating air pollution and improving mental health (Cohen-Cline *et al* 2015). A longitudinal study found that optimal physical activities can help improve sleep quality during pregnancy (Tan *et al* 2020). In a study with personal exposure assessment, results suggest that pregnant women living in greener areas were exposed to lower levels of indoor and outdoor air pollution and spent more time outdoors (Dadvand *et al* 2012). Another study revealed that during pregnancy, social cohesion has a mediation effect on mental health (Subiza-Pérez *et al* 2021). During the past decade, a growing number of epidemiological studies have been conducted, and their results suggest that there is a potential association between exposure to green space and birth outcomes such as birth weight (Zhan *et al* 2020, Hu *et al* 2021). Existing studies used the corresponding year or the entire pregnancy period to represent pregnancy exposure. However, pregnancy period is less than a calendar year, and fetal development is dynamic throughout the pregnancy. Pregnancy is a sensitive period during which intrauterine exposures can modulate fetal development and confer a long-lasting effect on the offspring (Workalemahu *et al* 2018). Meanwhile, greenness varies by season and is affected by the local climates. Exposure assessed at a fixed location without considering temporal variation, as conventionally done, cannot represent and be used to identify the critical exposure periods. Limited information is available for identifying the critical period of greenness exposure associated with birth outcomes in an intra-urban-suburban environment (Hu *et al* 2021). Research on the critical environmental exposure window during pregnancy has been underscored by studies focusing on air pollution (Chen *et al* 2021, Miron-Celis *et al* 2023). With regard to greenness exposure, a study from California suggests that green spaces may have a more potent protective association with preterm birth (PB) during the second trimester (Sun *et al* 2020). In contrast, a study from Tel Aviv highlighted the first trimester as a potential exposure window (Agay-Shay *et al* 2019). These findings demonstrate inconsistency and call for further validation, particularly in densely populated urban centers like Shanghai, where green spaces are a rare commodity. Furthermore, pinpointing the critical exposure window will help steer further research directions into the biological mechanisms influenced by greenness exposure during pregnancy. In this study, we aimed to evaluate the association of the urban residential surrounding greenness with birth weight, birthweight *Z*-score, small-for-gestational age (SGA) and large-for-gestational age (LGA) and test potential pathways through which greenness exerts beneficial effect on birth outcomes health through mediation analysis. Our research can add new scientific evidence to the literature on the association between residential greenness and birth outcomes by identifying critical exposure period during the pregnancy. In particular, by assigning greenness exposure to each period of preconception and three trimesters, our study could improve the understanding of the critical exposure time window that affects birth weight.

## 2. Method

### 2.1. Health baseline and birth outcomes data

**Figure 1.**
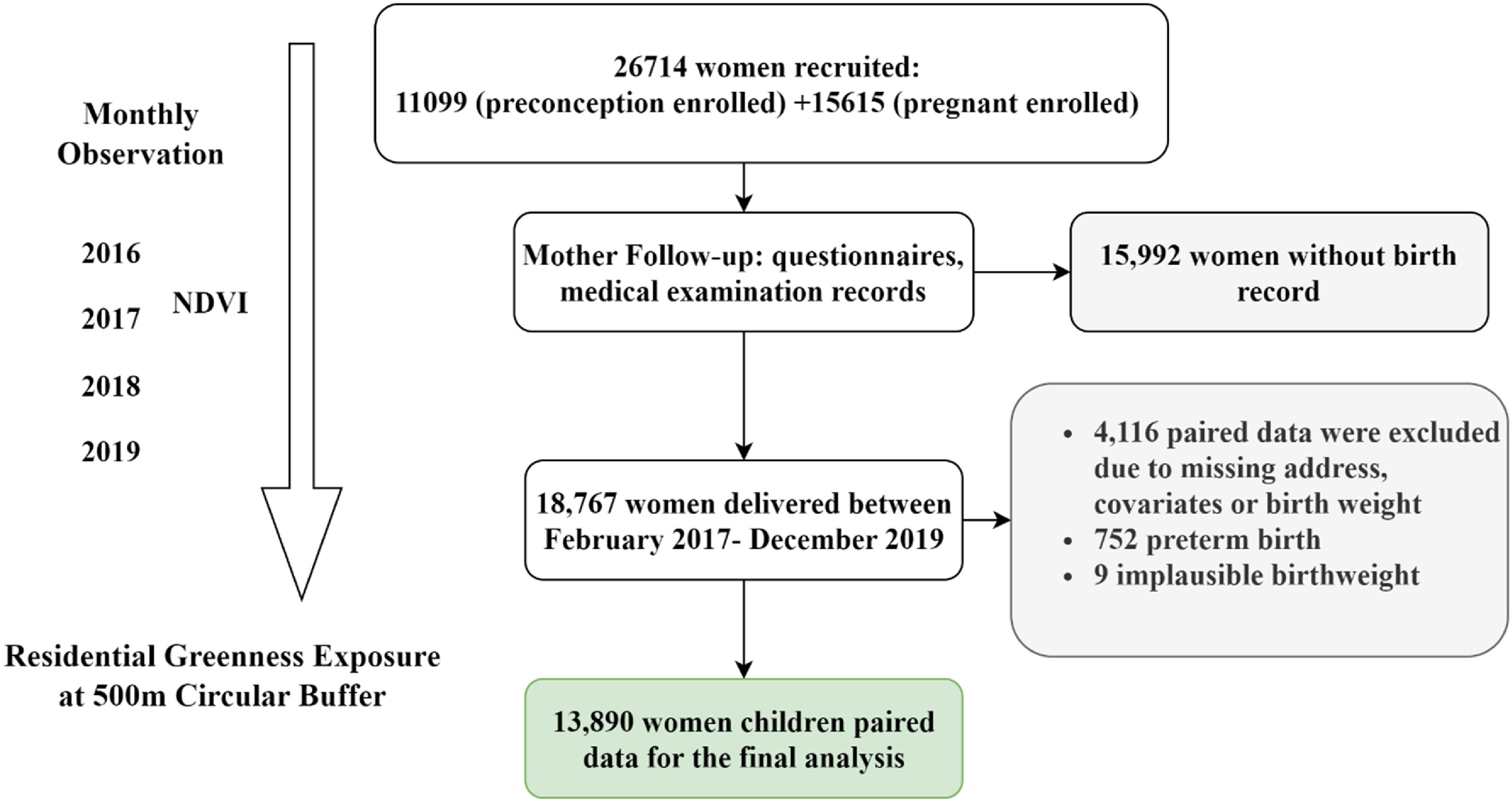
Flow diagram of the Growth and Air Pollution in Preconception (GAAP) cohort participant screening, showing the final sample size of 13 890 used in the main data analysis.

This Growth and Air Pollution in Preconception (GAAP) study utilized the birth outcome data based on an existing birth cohort of Shanghai Preconception Cohort (SPCC, NCT02737644) which enrolled *N* = 26 714 women during preconception (*n* = 11 099; 8045 preconception couples and 3054 single women) or early pregnancy (*N* = 15 615) at preconception or prenatal clinics from 28 maternity institutions in 10 districts of Shanghai, between March 2016 and December 2018. The SPCC study has been described in detail elsewhere (Wang *et al* 2019). We excluded 15 992 women without birth record, 4116 due to missing address, covariates, exposure assessment or child birthweight information, 752 children born preterm and 9 implausible birthweight (figure 1). The Ethics Committee of the Children’s Hospital of Fudan University (IRB number: 201649), Duke University (IRB number: 00000560) and University of Southern California (IRB number: HS-19-00306) approved this study.

In this study, data of key demographics at baseline was retrieved from the questionnaire that participants completed in the preconception clinics. Baseline demographical information included maternal age in years, ethnicity, education levels and employment. Lifestyle data including maternal smoking, exposure to secondhand smoke, nutrition supplementation information during the peri-conception period such as folic acid supplement was collected when participants visited the first antenatal care (∼14 weeks of gestational age). Additionally, we used the routine antenatal care electronic medical record data from the Maternal Clinic Antenatal Medical Record System (MCAMRS), which allowed to get access the information of gestational weight and height, last menstrual period, gestational week measured by ultrasound or last menstrual period. Based on the maternal height and weight, we calculated the maternal body mass index (BMI) at early pregnancy and further categorized into underweight (BMI < 18.5), normal weight (BMI ⩾ 18.5 kg m^−2^ and <24 kg m^−2^), overweight (BMI ⩾ 24 kg m^−2^ and <28 kg m^−2^), and obese (BMI ⩾ 28 kg m^−2^) referring to the definition of the Chinese population (Xi *et al* 2012). After children were born, we obtained the birth certificate information from the MCAMRS and retrieved information of delivery date, delivery mode, birthweight, child’s sex, birth defects and pregnancy complications if any. We calculated the gestational week at born and excluded PB with gestational weeks <37 weeks, we further categorized trimesters of pregnancy as the first trimester: 0–13 gestational weeks (day 0–day 91), the second trimester: 14–26 gestational weeks (day 92–day 182), and the third trimester: 27 gestational weeks (day 183) to the delivery.

In the analysis, birth outcomes included birth weight in grams, birthweight *z*-score and gestational age-specific birth weight categories including small for gestational age (SGA and LGA. The birthweight data was collected by measuring the newborn within 72 h after birth by a nurse in obstetrics in the MCAMRS system. The primary outcome was birth weight of full term (gestational age ⩾ 37 weeks). Additionally, we estimated the gender and gestational age-specific birth weight percentiles of each newborn using recorded birth weight, gestational age at delivery and reference percentile chart for Chinese representative children (Dai *et al* 2014). Based on the 10th and 90th percentiles cut-offs, we classified the gender and gestational age-specific birth weight into three categories: (1) SGA as less than 10th percentile, (2) normal for gestational age (NGA) as between 10th and 90th percentile, and (3) LGA as above 90th percentile.

### 2.2. Residential surrounding environment exposure

We conducted a buffer distance analysis to summarize the greenness and park features of the residential surrounding environment. To represent participants’ exposure to surrounding greenness, we used the NDVI, a satellite-based index. Specifically, we utilized the smoothed and gap-filled composite Moderate Resolution Imaging Spectroradiometer (MODIS) NDVI data and combined measurements from Terra and Aqua satellite instruments. When retrieving the data, we removed the negative values of NDVI to reduce the effects from water and cloud (Sharma *et al* 2022). The data was available at a spatial resolution of 250 m and temporal resolution of 16 d. To ensure comprehensive spatial coverage, we created circular buffers of 500 m radius around each participant’s residential address. This buffer size was selected based on two factors: (1) it is the most widely used and effective buffer size in greenness exposure studies related to birth outcomes (Hu *et al* 2021), and (2) it is centered around the dwelling, so participants can walk to greenspaces within 5–10 min. This range represents the neighborhood environment in which potential physical activities can take place (Labib *et al* 2020). For temporal coverage, we averaged the NDVI values by month and aggregated them over various pregnancy periods (preconception, and first, second, and third trimesters). We also determined the distance to the nearest park using point-of-interest data from the AMAP (http://m.amap.com) service. All locations were manually checked on the map, and we categorized addresses into two groups based on whether there was a park within a 500 m circular buffer. This distance represents the physical activity function of greenspace. We retrieved our satellite data from Google Earth Engine (https://earthengine.google.com/) using the geemap package (Wu 2020) and performed all geospatial calculations using Python (www.python.org/, version 3.88) and R (www.r-project.org/, version 4.13).

Additionally, we estimated ambient air pollution levels in this study based on residential addresses using the temporal-spatial model, (Xu *et al* ), and averaged PM_2.5_ and NO_2_ exposure over pregnancy periods for each participant as the greenness exposure assessment. The air pollution models provide PM_2.5_ and NO_2_ estimations at 3 day temporal resolution and 1 km spatial resolution in Shanghai between October 2012 and December 2019.

### 2.3. Covariates and statistical analysis

Potential confounders and effects modifiers were selected based on the previous studies. Biological variables recorded from the questionnaire are maternal age at birth, maternal BMI at first pregnancy, delivery mode, gestational week at delivery, parity, child’s sex and nutrition supplementation information during the periconception period such as folic acid supplement. Ambient temperature and pollution levels vary by season. Hence, we further controlled season of birth as Spring (March, April and May), Summer (June, July and August), Autumn (September, October and November), and Winter (December, January and February). Maternal social variables (educational levels and occupations), smoking history (yes or no), exposure to environmental smoke (yes or no) and delivery mode (vaginal delivery and Cesarean section). Educational levels include three categories: (1) high school and below, (2) college, and (3) postgraduates and above. Occupations includes seven categories of (1) manager (2) technician (3) entrepreneur (4) worker (5) farmer (6) self-employed, and (7) other. We further classified the occupations into three groups of group A (manager, technician, and entrepreneur), group B (worker, farmer, and self-employed), and group C (other or unemployed). Missing data of education levels, occupations, smoking history and environmental smoke exposure were coded as a separate category for these variables to incorporate all participants in the analysis. We did not consider altitude and slope in this study because Shanghai is an alluvial plain with an average altitude of 4 m.

**Figure 2.**
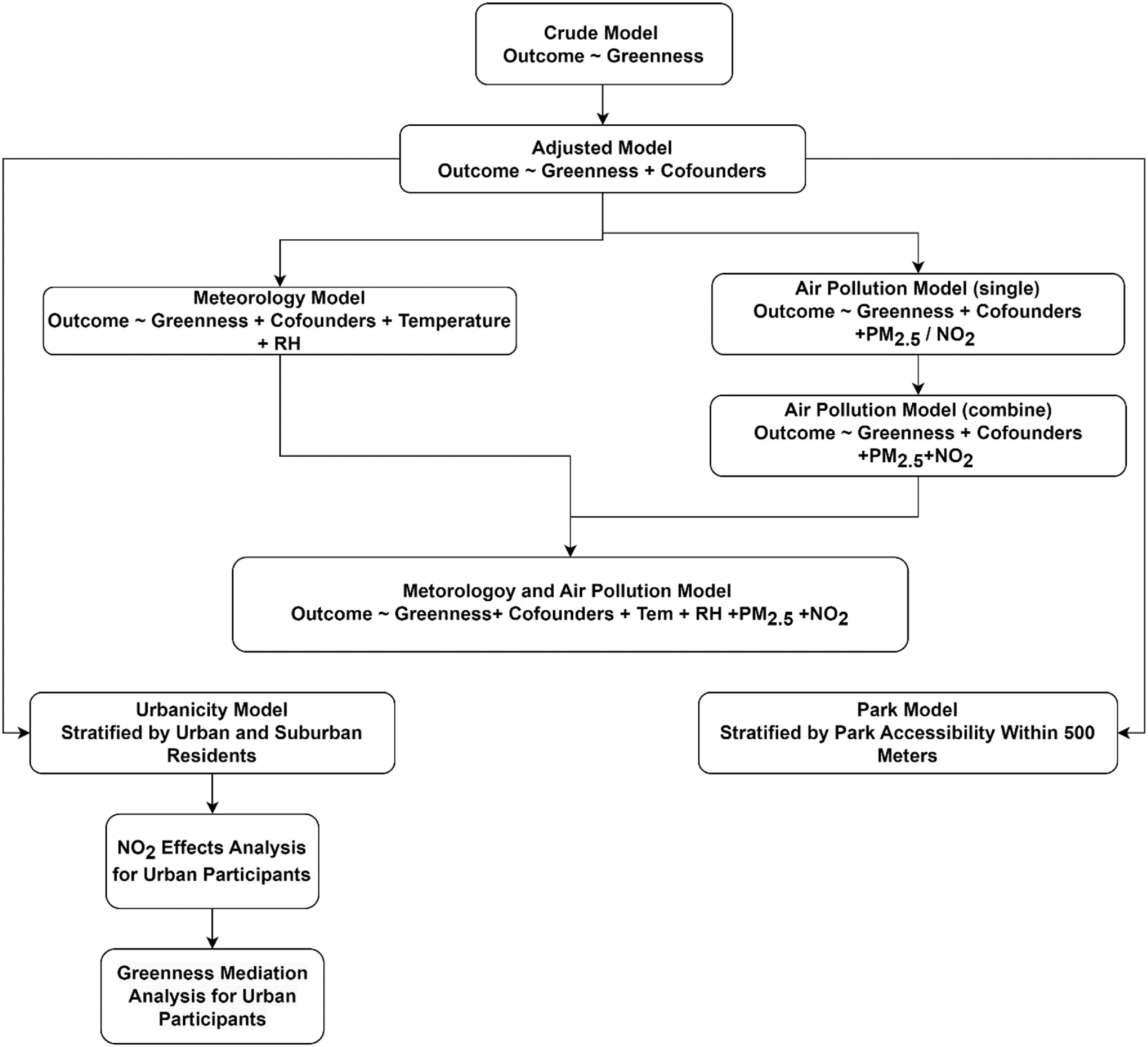
Flow diagram of statistical model analysis of controlling meteorology and air pollution variables.

First, we ran a basic model considering only exposure and outcomes. Subsequently, we investigated the potential exposure-response relationship between NDVI and the outcomes using the Generalized Additive Models (GAM). We employed multivariate linear regression models to investigate the association between environmental exposures and birth weight and *Z*-score. We used multivariate logistic regression models for SGA and LGA as categorical variables (figure 2). We performed our analysis based on a DAG (Directed Acyclic Graph) to depict the causal relationships between variables (figure S1).

We conducted a number of sensitivity analyses to test the robustness of our findings. Firstly, we tested the greenness association with birth outcomes by quartile of NDVI values through different stages of pregnancy. Secondly, we further controlled contemporaneous meteorological conditions (relative humidity and temperature) and air pollution (PM_2.5_ and NO_2_) during the whole pregnancy in the model. To test individual air pollutant’s effect, we only controlled one pollutant at a time in the model, as model PM_2.5_ and model NO_2_, respectively. Considering air pollution levels were potentially correlated with NDVI values during the same trimester due to seasonal effects, we further controlled the air pollution level throughout the entire pregnancy as well as the meteorological factors of the same pregnancy period across trimesters. We further stratified the participants by urbanicity (urban vs suburban) and park accessibility (park within 500 m buffer) and test the consistency of our findings of different residential environments. Urban area was defined as the area within outer circle (or ‘Waihuan’) highway.

To investigate whether NO_2_ levels mediate the association between greenness and birthweight during the second trimester (the period found to be most critical) and the whole pregnancy among participants who lived in urban areas, we conducted a mediation analysis using the ‘mediation’ package in R (Tingley *et al* 2014). We first fitted two regression models for each exposure period: the mediator model: A model predicting NO_2_ levels (M) based on greenness exposure (X) and any necessary control variables (C). The outcome model: A model predicting birthweight (Y) based on greenness exposure (X), NO_2_ levels (M), and any necessary control variables (C). Then we estimated the average causal mediation effects (ACME), average direct effects (ADE), total effects, and the proportion mediated for each exposure period. The ACME represents the indirect effect of greenness on birthweight through NO_2_ levels, while the ADE represents the direct effect of greenness on birthweight after accounting for NO_2_ levels. The total effect is the sum of the direct and indirect effects, and the proportion mediated represents the proportion of the total effect that is attributable to NO_2_ levels. The covariates adjusted for are the same as the main model of the adjusted model (figure 2). To estimate the uncertainty of the mediation effects, we employed the default nonparametric bootstrap method provided by the ‘mediation’ package. This method involves repeatedly resampling the data and re-estimating the mediation effects to construct empirical CIs. We generated 1000 bootstrap samples to calculate 95% CIs for the ACME, ADE, total effects, and proportion mediated.

\begin{align*}{\text{Mediator Model}}:{\text{ N}}{{\text{O}}_{\text{2}}} &amp; = \alpha + {\beta _1}{\text{X + }}{\beta _2}{\text{C + }}{\varepsilon _1}\end{align*}

\begin{align*}{\text{Outcome Model}}:{\text{Y }}\left( {{\text{Birthweight}}} \right) &amp; = \gamma + {\delta _1}{\text{X}} + {\delta _2}{\text{M}} + {\delta _3}{\text{C}} + {\varepsilon _2}.\end{align*}

Results were considered statistically significant at the *p* < 0.05 level (two-tail). Statistical analyses were conducted with R (www.r-project.org/ version 4.1.3).

## 3. Results

### 3.1. Characteristics of participants

Tables 1 and S1 (supporting information) provide the summary statistics of our study population. Our analysis included 13 890 paired women (mean age: 33.4, SD: 3.8) and their children (mean gestational age: 39.2 weeks, SD: 1.1 weeks). Over 80% of the participants had a college education or above, with more than half (58.2%) not reporting their employment status. The majority of participants (92.2% or 77.2%) did not smoke or were not exposed to environmental smoke during pregnancy, and all participants were married. Most women had a BMI below 24 kg m^−2^ when measured during the first antenatal care visit at around 14 weeks of gestational age. The children in our study were born between 2017 and 2019, with more than half (58.2%) born in 2018. The majority of births (30.5%) occurred in the fall. Folic acid supplementation was used by less than a third (29.0%) of the participants during preconception and early pregnancy. The average birth weight for full-term births was 3358.7 g (SD: 401.7 g).

**Table 1.** Summary of Study Participants’ Characteristics and Birthweight Outcomes.

Characteristic	Total (*N* = 13 890)
Birth Characteristics	
Sex of infant (# of births (%))	
Male	7883 (56.8%)
Female	6007 (43.2%)
Gestational age (weeks) (mean ± stdev)	39.2 ± 1.1
Birth weight (g) (mean ± stdev)	3358.7 ± 401.7
Small-for-gestational age, *n* (%)	803 (5.8%)
Large-for-gestational age, *n* (%)	1777 (12.8%)
Maternal characteristics	
Maternal Age (mean ± stdev)	33.4 ± 3.8
Age (# of prenatal record (%))	
20–25	108 (0.8%)
25–30	2746 (19.8%)
30–35	7465 (53.7%)
35–40	2924 (21.1%)
40–45	601 (4.3%)
45–50	44 (0.3%)
50–55	2 (<0.1%)
Marital status (# of births (%))	
Married	100%
Unmarried	0%
Educational level	
High school or less	1141 (8.2%)
College level	9494 (68.4%)
Above college	2290 (16.5%)
Missing	965 (6.9%)
Occupation	
Worker/Farmer/Self-employed/office clerk	1076 (7.7%)
Manager/Technician/Entrepreneur	4729 (34.0%)
Missing/unemployed	8085 (58.2%)
Parity (# of births (%))	
One	10 700 (77.0%)
Two	3135 (22.6%)
Three or above	55 (0.4%)
Active smoking during pregnancy, *n* (%)	
Yes	150 (1.1%)
No	12 813 (92.2%)
Missing	927 (6.7%)
Environmental smoking, *n* (%)	
Yes	2236 (16.1%)
No	10 717 (77.2%)
Missing	937 (6.7%)
Maternal first trimester BMI category, *n* (%)	
BMI < 18.5	1896 (13.7%)
BMI < 24	10 117 (72.8%)
BMI ⩽ 28	1529 (11.0%)
BMI > 28	348 (2.5%)
Folic acid supplementation during preconception and early pregnancy	
Yes	4024 (29.0%)
NO	9866 (71.0%)
Cesarean, *n* (%)	5406 (38.9%)

During pregnancy, more than half of the participants resided in urban areas (61.7%), consistent with the high population density in those areas (table S1; figure S2). The majority of participants (73.5%) did not reside within 500 m to a park. Participants had moderate levels of greenness exposure during pregnancy (NDVI mean: 0.3, SD: 0.1).

### 3.2. Air pollution exposure assessment and meteorological conditions

In table S1, we presented the predicted levels of PM_2.5_ and NO_2_ at residential address level and measured temperature and relative humidity represent the entire city for the preconception, 1st trimester, 2nd trimester, 3rd trimester and whole pregnancy averages, respectively. The mean concentrations of NO_2_ and PM_2.5_ at the participants’ residences throughout pregnancy were 37.6 *μ*g m^−3^ (SD: 3.6) and 42 *μ*g m^−3^ (SD: 6.3), respectively. These levels were above the WHO guidelines for PM_2.5_ annual concentration of 5 *μ*g m^−3^ and NO_2_ annual concentration of 10 *μ*g m^−3^. The correlations among air pollution, meteorological conditions and greenness throughout the pregnancy periods are included in figure S3. For instance, temperature is highly positively correlated with NDVI values showing a seasonal trend.

### 3.3. Association between greenness and birth outcomes

Table 2 shows that higher NDVIs were associated with higher birthweight and *Z*-scores during certain time periods of pregnancy. Specifically, the adjusted model estimates suggest that higher NDVI during the 2nd trimester and whole pregnancy is associated with higher birthweight, and higher NDVI during the 1st trimester, 2nd trimester, and whole pregnancy is associated with higher *Z*-scores. However, no significant associations were observed between exposure to greenness and the pregnancy outcomes of LGA and SGA during any period of pregnancy. Quartile greenness analysis also show that birthweight was significantly associated with greenness exposure during the 2nd trimester and whole pregnancy (table S2). We did not observe a nonlinear relationship between exposure to greenness and birthweight according to the GAM model results (table S3).

**Table 2.** Changes in birthweight (g), *Z*-score and odds ratios (ORs) of SGA and LGA per 0.1 unit increase in NDVI value within a 500 m circular buffer.

		NDVI (Crude Model)		NDVI (Adjusted Model)	
Outcome	Time period	Point estimate (95% CI)	*P* value	Point estimate (95% CI)	*P* value
Birthweight	Preconception	1.0 (−6.4,8.3)	0.801	5.2 (−2.9,13.4)	0.209
	1st Trimester	**10.1 (2.5,17.8)**	**0.009**	8.4 (0.0,16.8)	0.050
	2nd Trimester	**11.9 (4.2,19.7)**	**0.003**	**10.2 (1.8,18.5)**	**0.017**
	3rd Trimester	1.3 (−6.3,8.9)	0.739	6.6 (−1.6,14.9)	0.114
	Whole pregnancy	**12.1 (2.5,21.8)**	**0.013**	**10.1 (1.0,19.2)**	**0.030**
*Z*-score	Preconception	0.000 (−0.018,0.017)	0.961	0.015 (−0.006,0.036)	0.162
	1st Trimester	**0.024 (0.006,0.043)**	**0.009**	**0.022 (0.000,0.043)**	**0.049**
	2nd Trimester	**0.030 (0.012,0.049)**	**0.002**	**0.024 (0.003,0.045)**	**0.029**
	3rd Trimester	0.003 (−0.016,0.021)	0.765	0.016 (−0.005,0.037)	0.139
	Whole pregnancy	**0.030 (0.007,0.053)**	**0.012**	**0.025 (0.001,0.048)**	**0.039**
		OR (95% CI)	*P* value	OR (95% CI)	*P* value
SGA	Preconception	1.02 (0.94,1.10)	0.683	0.96 (0.87,1.05)	0.371
	1st Trimester	0.92 (0.85,1.00)	0.062	0.93 (0.85,1.03)	0.178
	2nd Trimester	**0.88 (0.81,0.96)**	**0.003**	0.93 (0.84,1.03)	0.146
	3rd Trimester	0.99 (0.91,1.08)	0.854	0.97 (0.88,1.07)	0.581
	Whole pregnancy	**0.9 (0.81,0.99)**	**0.038**	0.94 (0.84,1.04)	0.220
LGA	Preconception	1.02 (0.96,1.08)	0.523	1.05 (0.98,1.12)	0.150
	1st Trimester	1.05 (1.00,1.12)	0.067	1.03 (0.96,1.10)	0.371
	2nd Trimester	1.04 (0.98,1.10)	0.213	1.04 (0.97,1.11)	0.253
	3rd Trimester	0.99 (0.94,1.05)	0.766	1.05 (0.98,1.12)	0.141
	Whole pregnancy	1.04 (0.97,1.12)	0.248	1.05 (0.97,1.13)	0.199

Crude Model: no confounding variable is controlled. Adjusted Model: For birthweight outcome, models are controlled for maternal age, education, occupation, maternal BMI at first trimester, maternal parity, maternal smoking status, exposure to secondhand smoking, season of delivery, gestational age, children’s sex and folic acid supplement during the periconception; for *Z*-score, SGA and LGA outcomes, models controlled for same covariates except for children’s sex and gestational age. Bold texts in the table indicate significance, with a p-value < 0.05.

Our findings, presented in table 3, revealed that greenness exposure during the second trimester and throughout the entire pregnancy was consistently associated with birthweight and *Z*-score, even after accounting for meteorological conditions (Model Meteorology). However, we did not observe any significant association between greenness exposure and birth outcomes after controlling for air pollution levels (Model Air Pollution-combine). Then we controlled individual air pollutant of PM_2.5_ or NO_2_. The results indicate that PM_2.5_ was not affecting the greenness effects with minimal impact on the estimate and significance of greenness effects on the birth weight and *z*-score during the second trimester and whole pregnancy (Model PM_2.5_). However, after controlling NO_2_, the effect size of the association between greenness exposure during the second trimester and birth weight decreased from 9.8 grams (95% CI: 1.1–18.6) to 8.5 grams (95% CI: −0.3–17.3), and the association became non-significant (Model NO_2_). Finally, when controlling both air pollution and meteorology, a significant association only observed during the whole pregnancy (9.8 g, 95% CI: 0.1–19.5 g) (table S4). For sensitivity analysis, we only controlled the air pollution levels averaged during the whole pregnancy, we found a significant association between greenness during the second trimester (9.6 g, 95% CI: 0.4–18.7 g) and the entire pregnancy (9.8 g, 95% CI: 0.1–19.5 g) with birthweight (table S5). And, for *Z*-score, a significant association was observed only during the second trimester (0.024, 95% CI: 0.000–0.047) (table S5).

**Table 3.** Changes in birthweight (g), *Z*-score and odds ratios (ORs) of SGA and LGA per 0.1 unit of NDVI value of 500 m circular buffer increase controlling factors of meteorological conditions and/or air pollution levels.

		NDVI (Model Meteorology)		NDVI (Model Air Pollution-combine)		NDVI (Model PM_2.5_)		NDVI (Model NO_2_)	
Outcome	Time period	Point estimate (95% CI)	*P* value	Point estimate (95% CI)	*P* value	Point estimate (95% CI)	*P* value	Point estimate (95% CI)	*P* value
Birthweight	Preconception	6.4 (−2.3,15.0)	0.149	4.8 (−3.8,13.5)	0.276	5.9 (−2.3,14.2)	0.158	5.2 (−3.4,13.8)	0.238
	1st Trimester	8.1 (−0.7,17.0)	0.070	6.6 (−2.3,15.5)	0.148	7.8 (−0.8,16.4)	0.075	6.6 (−2.3,15.5)	0.148
	2nd Trimester	**9.8 (1.1,18.6)**	**0.027**	8.6 (−0.3,17.4)	0.058	**9.6 (1.1,18.2)**	**0.028**	8.5 (−0.3,17.3)	0.058
	3rd Trimester	8.1 (−0.6,16.7)	0.067	6.7 (−2.0,15.4)	0.132	6.8 (−1.6,15.2)	0.113	6.7 (−2,15.4)	0.133
	Whole pregnancy	**10.0 (0.8,19.2)**	**0.033**	9.1 (−0.5,18.7)	0.063	**9.9 (0.7,19)**	**0.034**	8.5 (−1,18)	0.080
*Z*-score	Preconception	0.015 (−0.007,0.038)	0.176	0.015 (−0.008,0.037)	0.197	0.017 (−0.005,0.038)	0.123	0.016 (−0.007,0.038)	0.172
	1st Trimester	0.021 (−0.002,0.043)	0.073	0.017 (−0.006,0.04)	0.139	0.02 (−0.002,0.042)	0.073	0.017 (−0.006,0.04)	0.139
	2nd Trimester	**0.024 (0.002,0.047)**	**0.033**	0.022 (−0.001,0.045)	0.057	**0.024 (0.002,0.046)**	**0.032**	0.022 (−0.001,0.045)	0.060
	3rd Trimester	0.021 (−0.002,0.043)	0.069	0.017 (−0.005,0.039)	0.136	0.017 (−0.004,0.039)	0.117	0.017 (−0.005,0.039)	0.136
	Whole pregnancy	**0.025 (0.002,0.049)**	**0.037**	0.022 (−0.002,0.047)	0.075	**0.024 (0.001,0.048)**	**0.042**	0.022 (−0.003,0.046)	0.079
		OR (95% CI)	*P* value	OR (95% CI)	*P* value	OR (95% CI)	*P* value	OR (95% CI)	*P* value
SGA	Preconception	0.93 (0.84,1.03)	0.157	0.95 (0.86,1.05)	0.321	0.95 (0.87,1.05)	0.316	0.95 (0.86,1.05)	0.301
	1st Trimester	0.94 (0.85,1.04)	0.220	0.93 (0.84,1.03)	0.177	0.92 (0.84,1.02)	0.122	0.93 (0.84,1.03)	0.184
	2nd Trimester	0.96 (0.86,1.06)	0.400	0.96 (0.87,1.07)	0.482	0.95 (0.86,1.05)	0.300	0.96 (0.87,1.07)	0.468
	3rd Trimester	0.97 (0.87,1.07)	0.493	0.99 (0.89,1.10)	0.830	0.99 (0.89,1.09)	0.780	0.99 (0.89,1.1)	0.828
	Whole pregnancy	0.94 (0.85,1.05)	0.292	0.95 (0.85,1.06)	0.328	0.94 (0.84,1.04)	0.236	0.95 (0.85,1.07)	0.410
LGA	Preconception	1.04 (0.96,1.11)	0.337	1.05 (0.98,1.13)	0.189	1.05 (0.98,1.12)	0.166	1.05 (0.98,1.12)	0.192
	1st Trimester	1.04 (0.97,1.11)	0.309	1.03 (0.96,1.11)	0.456	1.03 (0.96,1.1)	0.422	1.03 (0.96,1.11)	0.455
	2nd Trimester	1.07 (0.99,1.14)	0.081	1.05 (0.98,1.13)	0.144	1.05 (0.98,1.13)	0.148	1.05 (0.98,1.13)	0.154
	3rd Trimester	1.06 (0.99,1.14)	0.106	1.06 (0.98,1.13)	0.129	1.06 (0.99,1.13)	0.110	1.06 (0.98,1.13)	0.131
	Whole pregnancy	1.06 (0.98,1.14)	0.128	1.05 (0.97,1.13)	0.222	1.05 (0.98,1.13)	0.190	1.05 (0.98,1.14)	0.185

For birthweight outcome, models are controlled for maternal age, education, occupation, maternal BMI at first trimester, maternal parity, maternal smoking status, exposure to secondhand smoking, season of delivery, gestational age, children’s sex and folic acid supplement during the periconception; for *Z*-score, SGA and LGA outcomes, models controlled for same covariates except for children’s sex and gestational age. Air pollution level of PM_2.5_ or NO_2_ and meteorological factors of temperature and relative humidity of the same period are controlled. Bold texts in the table indicate significance, with a p-value < 0.05.

**Figure 3.**
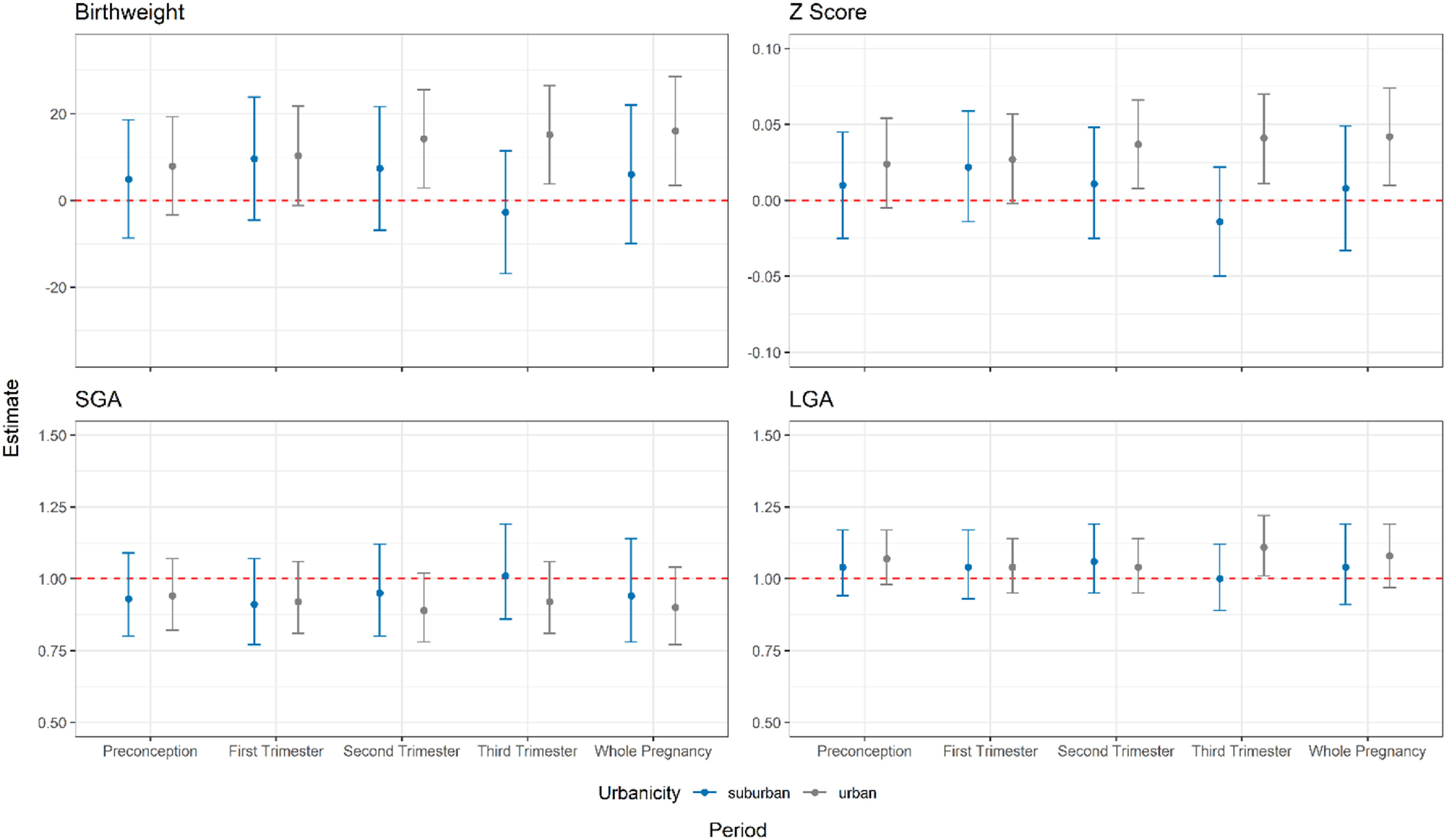
Changes in birthweight *Z*-score, birthweight, and odds ratios (ORs) of SGA and LGA associated with 0.1 unit increase of NDVI value within 500 m circular buffer during preconception and gestational periods of participants living in urban and suburban areas. (For birthweight outcome, models are controlled for maternal age, education, occupation, maternal BMI at first trimester, maternal parity, maternal smoking status, exposure to secondhand smoking, season of delivery, gestational age, children’s sex and folic acid supplement during the periconception; for *Z*-score, SGA and LGA outcomes, models controlled for same covariates except for children’s sex and gestational age).

In the stratified analysis by urbanicity (figure 3), we found a significant association between greenness exposure and birthweight and *Z* score during the 2nd trimester, 3rd trimester and whole pregnancy only for the participants who lived in the urban areas (table S6). Specifically, a 0.1 unit increased in NDVI value was associated with an increase in birthweight of 14.2 g (95% CI: 2.9–25.5 g), 15.2 g (95% CI: 3.8–26.5 g) and 16.0 g (95% CI: 3.5–28.5) and in *Z*-score of 0.037 (0.008–0.066), 0.041 (0.011–0.070) and 0.042 (0.010–0.074), in 2nd trimester, 3rd trimester and the whole pregnancy, respectively. We also find a positive association between LGA and greenness exposure during the third trimester. For the urban participants, we also observed per 10 *μ*g m^−3^ increase of exposure to NO_2_ during the 2nd and whole pregnancy trimester associated with a decrease in birthweight of 15.7 g (95% CI: 3.9–27.4 g, *P*-value = 0.009) and 23.7 g (95% CI: 7.6–39.7 g, *P*-value = 0.004) (table S7). We performed NO_2_ mediation analysis because we did not observe any significant associations for PM_2.5_. Results from this analysis suggested a potential mediating effect of NO_2_ in the relationship between greenness and birthweight during the 2nd trimester of pregnancy among urban participants (tables S8, S9 and S10). The mediation analysis for participants living in urban areas showed that 22.7% (95% CI: −0.3%–98%, *p* = 0.054) of the association between greenness and second trimester birthweight and 16.8% (95% CI: 4.0%–84%, *p* = 0.018) of the association between greenness and whole pregnancy birthweight were mediated by NO_2_ (table S9). The remaining direct effect of greenness on birthweight during the second trimester was not statistically significant (*p* = 0.056), while the direct effect for the whole pregnancy was significant (*p* = 0.046).

**Figure 4.**
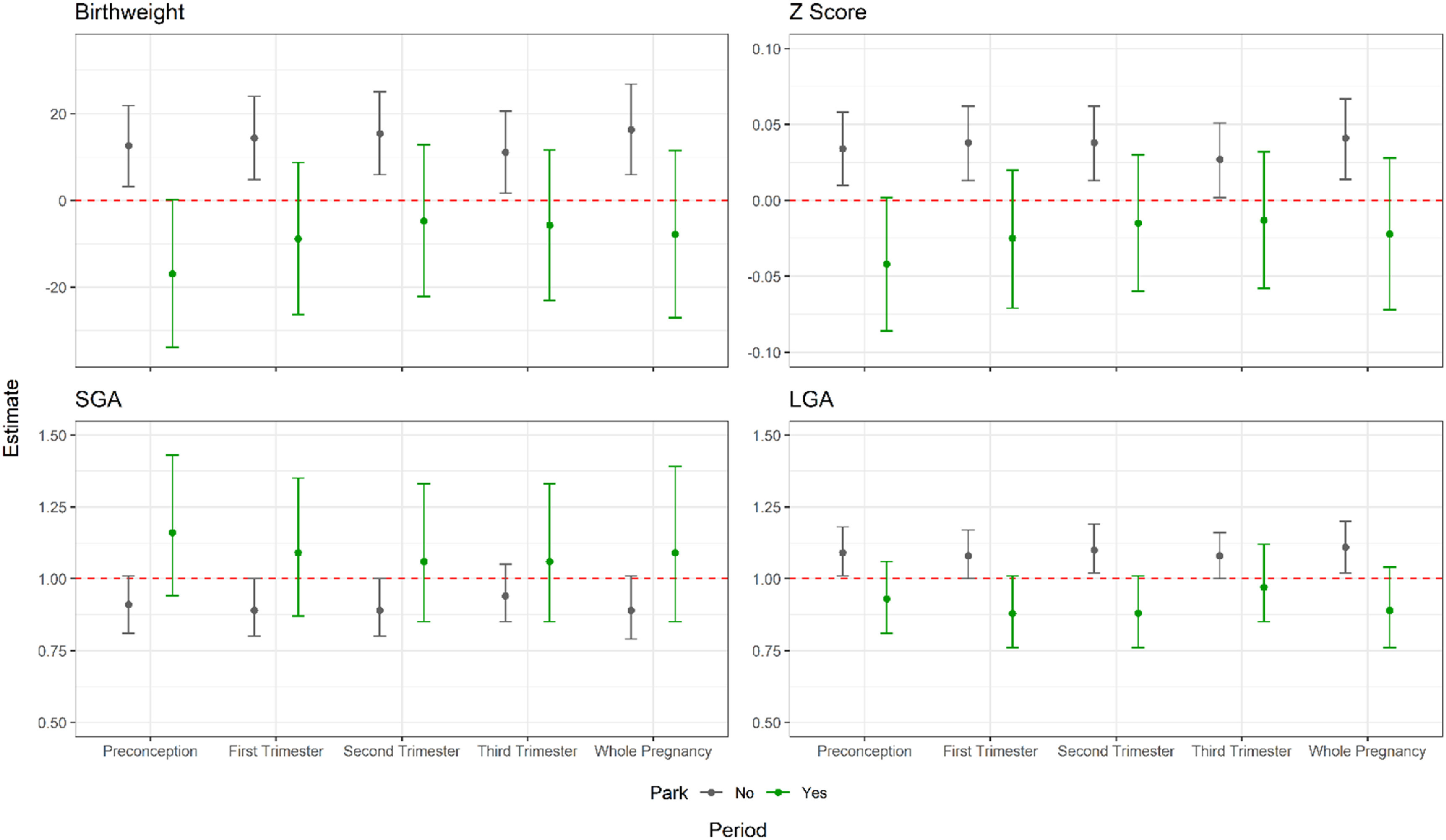
Changes in birthweight *Z*-score, birthweight, and odds ratios (ORs) of SGA and LGA associated with 0.1 unit increase of NDVI value within 500 m circular buffer during preconception and gestational periods of participants living within 500 m of a park. (For birthweight outcome, models are controlled for maternal age, education, occupation, maternal BMI at first trimester, maternal parity, maternal smoking status, exposure to secondhand smoking, season of delivery, gestational age, children’s sex and folic acid supplement during the periconception; for *Z*-score, SGA and LGA outcomes, models controlled for same covariates except for children’s sex and gestational age.).

Figure 4 presented the results of stratified analysis of park accessibility (whether they had access to a park within 500 m) based on adjusted model. The results revealed a significant association between greenness exposure during all pregnancy periods and the birthweight and *Z*-score of children who had no park within 500 m (figure 4 and table S11). Among this group, the largest effect was observed for the second trimester, with a 15.4 g increase in birth weight (95% CI: 5.9–25.0 g) and a 0.038 increase in *Z*-score (95% CI: 0.013–0.062), respectively. In contrast, the smallest effect was seen during the 3rd trimester, with a 11.1 g increase in birth weight (95% CI: 1.7–20.6 g) and a 0.027 increase in *Z*-score (95% CI: 0.002–0.051). Additionally, we found that higher greenness during the 1st and 2nd trimesters were significantly associated with the lower risk of SGA, respectively.

## 4. Discussion

In this study, we investigated the associations between greenness and birth outcomes in urban and suburban Shanghai, a mega metropolitan area in China. It is the first study to investigate the association between greenness environment and birth weight outcomes segmented by pregnancy time period, including preconception and the three trimesters. Our findings indicate that residential greenness exposure within a 500 m radius was associated with birthweight *Z*-scores and birthweights both during the entire pregnancy and the 2nd trimester. Our study identified a potential critical window of greenness exposure and added new evidence on the association of exposure to residential greenness with birth outcomes using a longitudinal approach throughout pregnancy. The results of this study also suggest a mediation effects of NO_2_ toward greenness exposure effects during the 2nd trimester among the urban participants.

We found that an increase in greenness (0.1 unit of NDVI) during the entire pregnancy was associated with a 10.1 g (95% CI: 1.0–19.2 g) increase in birthweight among full-term birth children. Our results are consistent with the findings of a previous systematic review and a meta-analysis that suggested a potential positive association between residential greenness and higher birth weights, ranging from 7.99 g (95% CI: 4.29–11.70) to 15.35 g (95% CI: 11.41–19.29) per 0.1 NDVI increase (Hu *et al* 2021). Comparing to the study conducted in the same country of China, Xiao *et al* (2022) found that residential greenness was associated with an increased birth weight by 35.4 g (95% CI: 13.2, 57.7) per 0.1 NDVI increase in urban areas of Maoming, Guangdong province. The higher effect size of the Maoming study may be due to the inclusion of PBs in the final analysis. In contrast, we only included full-term births in our analysis. Also, Guangdong province is in further south to Shanghai city of this study, compared to Xiao *et al* (2022)’s study of the same buffer size (500 m), participants in that study exposed to an average NDVI of 0.42 (SD: 0.13) which is higher than the averaged NDVI 0.3 (SD: 0.1) of this study.

Based on our analysis of exposure windows, we found that the 2nd trimester was most critical for the effect of greenness exposure on birthweight outcomes. However, there are only a limited number of studies that have investigated the critical time windows of greenness exposure during pregnancy. For example, Sun *et al* (2020) found that the 2nd trimester had a stronger protective association with PB. While Agay-Shay *et al* (2019) identified the 1st trimester (for birthweight and LBW) and 2nd trimester (for very preterm deliveries) as critical greenness exposure time windows. In Agay-Shay *et al* (2019) study, the beneficial association between birthweight and greenness exposure was observed for all trimesters, with the strongest association during the 1st trimester (3rd/1st tertile: increase in mean birthweight by 16.4 g (95% CI: 7.8, 25.0). On the other hand, Cusack *et al* (2017) found no significant association or critical exposure window between greenness and birthweight after adjusting for individual characteristics, especially race and ethnicity. The inconsistent findings regarding the critical greenness exposure time window during pregnancy could be explained by the varying characteristics of greenness in different urban areas and countries, as well as differences in the study settings and populations (Hu *et al* 2021). Additionally, cultural factors could also play a role in determining the impact of greenness exposure on birth outcomes. Women from different cultures may have different behaviors and attitudes toward pregnancy, especially during the 1st trimester when they may experience physical discomfort, such as nausea and vomiting. For example, Chinese women are often advised to be less active during the 1st trimester (Zhou *et al* 2022), which could limit their exposure to greenness. A cross-sectional study on physical inactivity status among Chinese pregnant women suggested that the likelihood of physical activity increased as pregnancy progressed from the 1st trimester to the second and 3rd trimesters (Zhou *et al* 2022). Meanwhile, a meta-analysis of 102 studies incorporating 221 974 women from 34 countries found that the prevalence for self-reported anxiety symptoms was 24.6% in the third trimester which is higher than the other two semesters (Dennis *et al* 2017). The physical inactivity during the first trimester, coupled with the added burden of anxiety during the third trimester, may make moderate exposure to greenness during the second trimester particularly beneficial for birthweights. For instance, a study on a birth cohort of 11 292 individuals in China found that higher intensity levels of physical activity during pregnancy were associated with an increase in birthweight *z*-scores. This highlights the need to identify critical exposure time windows for greenness during pregnancy to improve birth outcomes.

Comparing studies of a similar design from different countries or regions presents inherent challenges. Greenness or green space varies significantly across the globe, influenced not just by tangible factors (Wright Wendel *et al* 2012, Wu *et al* 2019) like geography, urban planning policies, types of vegetation, and ecological habitats, but also by intangible aspects like local perceptions and behaviors (Wan and Shen 2015). For example, research on greenness effects on birth outcomes from Texas highlighted how differences in vegetation, trees, and general green spaces might explain divergent findings when juxtaposed with studies from other regions (Cusack *et al* 2017). Our investigation was set in a densely populated Asian city with a pronounced demand for urban greenness (Wu *et al* 2019).

We observed that participants living in urban areas showed a significantly greater effect compared to those living in suburban areas. We also found that participants who had no access to park within 500 m from their residence may benefit more for a unit increase in greenness. One explanation is so-called saturation or adaptation phenomenon, as NDVI values were higher for suburban residents who would be less responsive on a per-unit NDVI incremental basis (figure S4).

Our finding supports the urban planning policy of Shanghai that urban areas should develop green infrastructure for recreational purposes. From 2000 to 2016, the Shanghai government committed to increasing urban greenery by constructing green spaces in the central city, in line with Shanghai’s Five-Year Plans for ecological and environmental protection (Wu *et al* 2019, Zhong *et al* 2019). For example, the area of green spaces in the Shanghai city center increased from 2.6 km^2^ (0.9%) in 2005 to 26.1 km^2^ (9.0%) in 2015. By the start of our study in 2016, Shanghai had constructed large public green spaces in the central city thanks to these plans (Wu *et al* 2019). We also found that greenness had a greater association with birthweights and *Z*-scores when there was no park within 500 m. This evidence may indicate that the immediate surroundings of the residence are more important. In Chinese cities, the dominant type of residence is gated high-rise residential community or ‘Xiaoqu,’ (figure S5) which typically contains a large area of green space and/or recreational facilities for activities purposes. In addition, some parks in Shanghai require payment for access, which could limit local residents to visit and enjoy these public spaces. Public policies should be designed to maximize the use of green parks, especially by susceptible people such as pregnant women.

Current evidence is inconclusive regarding the adverse association between NO_2_ and decreasing birthweight, as only one European meta-analysis of nine studies has shown that birth weight decreases with a 10 *μ*g m^−3^ increase in NO_2_ (pooled beta = −13.63, 95% CI (−28.03, 0.77)) (Simoncic *et al* 2020). However, previous studies assessing greenness exposure (Hu *et al* 2021) lacked temporal resolution to identify the inverse relationship between greenness and air pollution due to seasonal effects, as they used exposure during the entire pregnancy or did not control for temporal changes in air pollution levels. Our study used trimester-wise analysis to explore the effects of greenness while considering temporal correlation between air pollution and greenness. After controlling for NO_2_, the greenness effect disappeared, and both PM_2.5_ and NO_2_ were found to be moderately correlated with NDVI during the 2nd trimester (Pearson correlation coefficient *r* = −0.49 and −0.55, respectively). In the stratified analysis of urban participants (*n* = 8575), a 10 *μ*g m^−3^ increase in NO_2_ exposure during the second trimester was associated with a decrease in birth weight of 15.7 g (95% CI: 3.9–27.4 g). Our mediation analysis suggests that NO_2_ may be a mediator of effects of greenness exposure among urban participants during the second trimester (*p*-values for ACME and proportion mediated were close to 0.05). However, it does indicate that NO_2_ is a potential mediator (*p*-value = 0.046) among urban participants during the whole pregnancy, referring to the ACME (2.7 g 95% CI: 0.8–4.7 g, *p*-value = 0.01) and proportion of mediation (16.8% 95% CI: 4% -84%, *p*-value = 0.018). In urban areas, traffic is typically the primary source of NO_2_, which typically reaches background levels at a distance of approximately 380 m from the road (Karner *et al* 2010). We used a buffer size of 500 m for the greenness analysis, which is close to the decreasing distance range of NO_2_ and explains the mediation effects and moderate correlation between greenness and NO_2_ concentrations. We did not observe any mediation effects of greenness exposure via PM_2.5_, likely because PM_2.5_ may impact birth outcomes in a different time window. This became evident when we examined the specific effects of NO_2_ and PM_2.5_ separately in our recent study using the same dataset (Liao *et al* 2023). More studies on NO_2_ and greenness mediation in metropolitan areas are suggested to further validate these findings.

While there exists a limited body of research revealing the biological mechanisms linking exposure to greenness, existing studies have posited DNA methylation (Xu *et al* 2021a, 2021b, Jeong *et al* 2022), blood glucose levels (Liao *et al* 2019) and glycolipid metabolism (Yu *et al* 2023) as potential biological mechanisms. For instance, a study involving 479 Australian women found that exposure to greenness was associated with signs of slower biological aging (Xu *et al* 2021b). Another study focused on maternal exposure to greenness during pregnancy and discovered a correlation with cord blood DNA methylation (Alfano *et al* 2023). Moreover, there’s evidence suggesting that exposure to green spaces might enhance glucolipid metabolism, potentially through an increase in vitamin D levels (Yu *et al* 2023). Notably, a prospective birth cohort study highlighted that maternal glucose levels, taken between 24 and 28 weeks of gestation, had an inverse relationship with residential greenness exposure (Liao *et al* 2019).

We postulate that these biological mechanisms might be interconnected with pathways related to greenness that encompass three primary functions: Harm Reduction (e.g. decreasing exposure to air pollution, noise, and heat), Restorative Functions (e.g. attention restoration and physiological stress recovery), and Capacity Building (e.g. promoting physical activity and bolstering social cohesion) as summarized by (Markevych *et al* 2017). If a pathway specifically involves an agent of harm, the biological mechanism might be directly associated with that particular agent. For instance, the effects of PM_2.5_ air pollution could impact the respiratory and cardiovascular systems via inflammation and oxidative stress (Thangavel *et al* 2022). Conversely, if the pathway primarily involves capacity-building, the mechanism could potentially be tied to mental or neurological functions such as stress (Triguero-Mas *et al* 2017). Finally, we did observe the inconsistent effects comparing to different buffer sizes (table S12), which is consistent to the previous studies (Labib *et al* 2020, Hu *et al* 2021). Therefore, their distinctive pathway and biological mechanism needs further investigation. There are several limitations in our study. Firstly, we did not have detailed information on the activities of pregnant women, which may have resulted in misclassification of greenness exposure. Secondly, we did not control for community variables, which may have led to issues with spatial autocorrelation. This is particularly relevant when participants live in close proximity to one another and may be exposed to similar levels of greenness and air pollution. Additionally, we experienced a loss to follow-up for 15 992 women who lacked birth records. Possible reasons for these missing records include infertility (16.95%, *n* = 2177 in Shanghai) as reported by Zhu *et al* (2022), and pregnancy loss (ranging between 10% and 24%) as indicated by Jiang *et al* (2023). The impact of this loss to follow-up on greenness effects remains uncertain. Finally, due to the use of a spatiotemporal model with a spatial resolution of 1 km for NO_2_ and PM_2.5_, we were unable to explore the effects of NO_2_ beyond the range of approximately 380 m from a road, where it typically degrades to background levels. These limitations should be considered when interpreting our results and suggest opportunities for future research to improve our understanding of the relationships and interactions among greenness, air pollution, and pregnancy outcomes.

## Data Availability

The data cannot be made publicly available upon publication due to legal restrictions preventing unrestricted public distribution. The data that support the findings of this study are available upon reasonable request from the authors.
